# Clinicopathological and Prognostic Significance of Survivin Over-Expression in Patients with Esophageal Squamous Cell Carcinoma: A Meta-Analysis

**DOI:** 10.1371/journal.pone.0044764

**Published:** 2012-09-27

**Authors:** Chunguang Li, Zhigang Li, Maoling Zhu, Tiejun Zhao, Ling Chen, Weidan Ji, Hezhong Chen, Changqing Su

**Affiliations:** 1 Department of Thoracic Surgery, Changhai Hospital, The Second Military Medical University, Shanghai, China; 2 Department of Gastroenterology, The First People's Hospital of Shanghai, Shanghai Jiao Tong University, Shanghai, China; 3 Department of Molecular Oncology, Eastern Hepatobiliary Surgical Hospital and Institute, The Second Military Medical University, Shanghai, China; University of Patras, Greece

## Abstract

**Background:**

The prognostic significance of survivin for survival of patients with esophageal squamous cell carcinoma (ESCC) remains controversial. Thus, meta-analysis of the literatures was performed in order to demonstrate its expression impact on ESCC clinicopathological features and prognosis.

**Methodology:**

Relevant literatures were searched using PubMed, EMBASE and Medline Databases. Revman5.0 software was used to pool eligible studies and summary hazard ratio (HR). Correlation between survivin expression and clinicopathological features of ESCC was analyzed.

**Principal Findings:**

Final analysis of 523 patients from 7 eligible studies was performed. Combined HR of survivin location in nuclei suggested that survivin expression has an unfavorable impact on ESCC patients' survival (n = 277 in 3 studies; HR = 1.89, 95% CI: 1.45–2.96; Z = 4.69; *P*<0.0001). Nevertheless, combined HR of survivin location in cytoplasm displayed that survivin expression has no significance for prognosis of ESCC patients (n = 113 in 2 studies; HR = 0.96, 95% CI: 0.96–5.69; Z = 0.04; *P* = 0.97); Combined odds ratio (OR) of survivin location in cytoplasm indicated that survivin expression is associated with ESCC advanced stage (n = 113 in 2 studies; OR = 0.36, 95% CI: 0.14–0.93; Z = 2.10; *P* = 0.04). Whereas, combined OR of survivin location in nuclei exhibited that survivin over-expression has no correlation with cell differentiation grade, lymph node status, depth of invasion, stage, and metastasis of ESCC.

**Conclusions:**

This study showed that survivin expression detected by immunohistochemistry seems to be associated with a worse prognosis of ESCC patients. Survivin subcellular location may be an important factor impacting on ESCC development. Larger prospective studies should be performed to evaluate the status of survivin in predicting prognosis of patients with ESCC.

## Introduction

Esophageal squamous cell carcinoma (ESCC) is the leading cause of cancer death worldwide. Despite advances in treatment, the benefit of surgical resection combination with chemotherapy or radiotherapy is not satisfactory. The prognosis of ESCC patients is still poor and the 5-year overall survival (OS) rate is only 20% to 30% [1]. Therefore, it is very important to search for biological markers, which can diagnose cancer as early as possible, estimate reaction to chemotherapy or radiotherapy in those patients with ESCC, and predict OS of patients undergoing treatment. As we know, an ideal tumor molecular marker can help us to assess prognosis and set up reasonable treatment. For example, alpha fetoprotein (AFP) is widely used for hepatocellular carcinoma in diagnosis and treatment. Nevertheless, no specific molecular marker can be used in ESCC routinely.

Induction of apoptosis is the main molecular mechanism of chemo- and radiotherapy to kill cancer cells [2]. Apoptosis inhibitory genes with certain activity in human cancers functions to promote cancer carcinogenesis and formation. Recently, survivin, an identified apoptosis inhibitor, was found to be expressed in malignancies and fetal tissues, but not in normal adult tissues [3]. Its over-expression was linked with poor prognosis in many cancers, such as non-small cell lung cancer [4], breast cancer [5], bladder cancer [6], liver cancer [7], pancreatic cancer [8]. Accordingly, survivin may become an important prognosis biomarker in human tumors. Survivin regulates the essential cellular processes of inhibiting apoptosis and promoting cell proliferation by controlling a series of downstream apoptosis genes, caspase-3 and caspase-7, leading to unresponse to apoptosis stimulus signals in cancer cells [9], which is one of the most important molecular mechanisms for drug resistance [10]. In addition, survivin is predominantly upregulated during the G2-M phase with a cell cycle dependent manner by the activation of cell cycle homology region within the promoter, suggesting that survivin can help cancer cells to overcome G2-M checkpoint to promote cell infinite proliferation [10].

**Figure 1:**
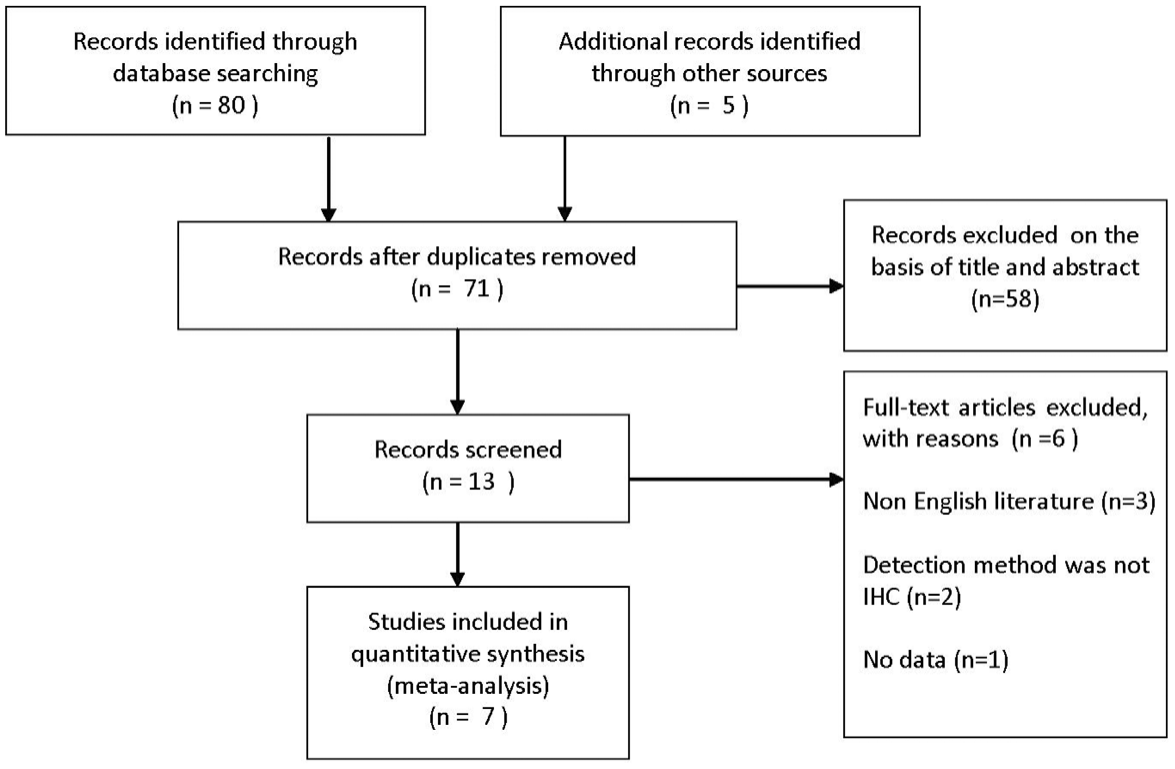
Flow diagram of study selection.

**Table 1:** Characteristics of enrolled studies.

First author	Year	Patient number	Stage		Gender		Median age	Clinicopathological features	Survivin effect on survival	Patients number received adjuvant therapy
			I–II	III–IV	M	F				
Grabowski	2003	84	31	53	60	24	56.8	D,LN,T,M,S	Yes	–
Dabrowski	2004	42	3	39	38	4	58.36	D,LN,T,M,S	Yes	No
Mega	2006	122	75	47	105	17	62.3	D,LN,T,M,S	Yes	Yes
Rosato	2006	56	23	33	49	7	62	D,LN,T,S	Yes	–
Takeno	2010	71	37	34	63	8	63.8	D,LN,T,M,S	Yes	–
Hsu	2009	46	29	17	43	3	61.4	D,LN,T,M,S	Yes	–
Zhu	2011	102	43	59	75	27	66	LN,T,M,S	Yes	Yes

D, histologic differentiation; LN, lymph node metastasis; T, depth of tumor invasion; M, metastasis; S, stage.

Survivin over-expression is associated with worse OS, lymph node metastasis and occurrence in majority of cancers. Generally, studies about the prognostic significance of survivin are comparatively few in ESCC. Some results are not entirely consistent. Thus, it is necessary to analyze the data of survivin in ESCC to reach a reasonable conclusion at present, and examination of survivin expression in ESCC after operation can help us to indentify high risk population of patients with poor prognosis.

**Table 2:** Immunohistochemical technique used in these studies.

First author	Antibody source	Dilution	Counting method	Definition of survivin positive
Grabowski	Novus Biologicals	0.25µg/ml	Percentage of positive cells	>5%
Dabrowski	Santa Cruz	1∶20	Percentage of positive cells	>5%
Mega	San Antonio	1∶20	Combination of staining intensity score and percentage of positive cells	>1 point score
Rosato	Santa Cruz	1∶80	Percentage of positive cells	>20%
Takeno	San Antonio	1∶20	Combination of staining intensity score and percentage of positive cells	>10% (nuclei); >50% (cytoplasm)
Hsu	R & D	1∶100	Combination of staining intensity score and percentage of positive cells	>175 point scores
Zhu	Santa Cruz	1∶100	Combination of staining intensity score and percentage of positive cells	>0 point score

In this study, we made a meta-analysis to investigate survivin expression in ESCC specimens and its association with surgical outcome of ESCC patients. The results showed that survivin expression is associated with ESCC clinicopathological features and patients' prognosis. The meta-analysis will help us to design better adjuvant therapy and give closer follow-up for the patients with survivin over-expression. In addition, better understanding of survivin expression and function in ESCC that distinguish from normal esophageal epithelia is also beneficial for potential target therapy of ESCC patients.

**Figure 2:**
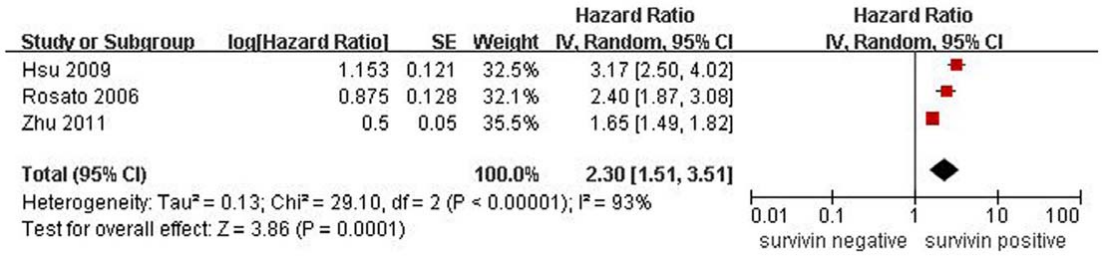
Forest plot of Hazard ratio (HR) for survival of ESCC patients. Survivin subcellular location was not clear in the 3 studies, the combined HR demonstrated that over-expression of survivin in ESCC was associated with patients' worse prognosis.

**Figure 3:**
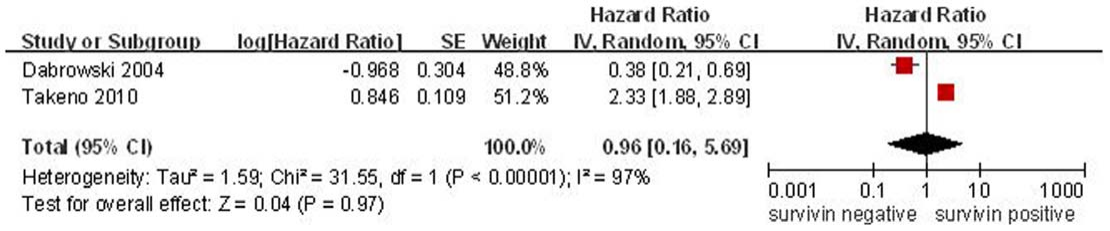
Forest plot of Hazard ratio (HR) for survival of ESCC patients. The combined HR demonstrated that cytoplasmic expression of survivin was not associated with prognosis of ESCC patients.

**Figure 4:**
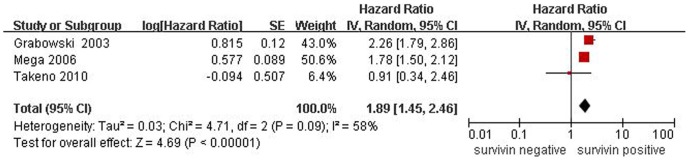
Forest plot of risk HR for survival of ESCC patients. The combined HR demonstrated that nuclear expression of survivin was associated with poor prognosis of ESCC patients.

**Figure 5:**
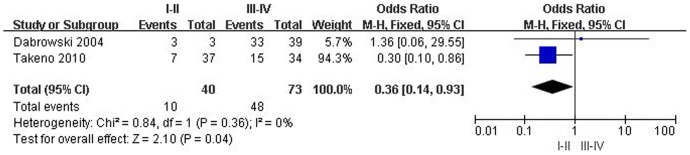
Odds ratio (OR) and 95% confidence interval (CI) in two studies evaluating the relationship between survivin over-expression and ESCC stage. OR<1 implied that survivin over-expression in cytoplasm was lower in patients with stage I/II than that with stage III/IV.

**Table 3:** Meta-analysis about survivin expression in ESCC cytoplasm.

Clinicopathological features	N	Cases	OR	95% CI	*P* value for OR	*P* value for heterogeneity
Differentiation grade	2	113	0.47	0.05–4.55	0.52	0.02
Lymph node status	2	113	0.53	0.21–1.32	0.17	0.17
Stage	2	113	0.36	0.14–0.93	0.04	0.36
Metastasis	2	113	1.09	0.39–3.06	0.87	0.48

N: number of studies; OR: odds ratio; CI: confidence interval.

**Table 4:** Meta-analysis about survivin expression in ESCC nuclei.

Clinicopathological features	N	Cases	OR	95% CI	*P* value for OR	*P* value for heterogeneity
Differentiation grade	2	150	1.92	0.73–5.05	0.19	0.39
Lymph node status	3	276	0.51	0.14–1.87	0.31	0.02
Depth of invasion	3	276	1.05	0.24–4.65	0.95	0.009
Stage	3	276	0.58	0.16–2.08	0.40	0.02
Metastasis	2	193	0.93	0.41–2.15	0.87	0.10

N: number of studies; OR: odds ratio; CI: confidence interval.

## Methodology

### Literature search

PubMed and Medline were searched for articles relating to survivin and ESCC from 1997 to March 2012. The following MESH headings key words and text words were used: (1) esophagus or esophageal or oesophagus or oesophageal, and cancer or tumor or neoplasm or carcinoma; (2) survivin or BIRC5. The references of articles and reviews were also manually searched for additional studies.

### Inclusion and exclusion criteria

We collected all eligible articles about relationship between survivin and clinicopathological features or OS in ESCC in this meta-analysis. All retrieved articles were carefully scanned to indentify some potential relevant reports. The deadline of the included articles was March 1, 2012. The study included in our meta-analysis should meet the following inclusion criteria as follows: (1) survivin expression evaluated in the primary ESCC tissues; (2) relationship demonstrated between survivin expression and ESCC clinicopathological parameters or prognosis; (3) survivin expression examined by immunohistochemistry; (4) articles published as a full paper in English; (5) studies provided sufficient information to estimate hazard ratio (HR) and 95% confidence interval (CI); (6) If multiple studies investigated the same patients or potential overlapping patients, only the most complete single study was selected. The exclusion criteria were as follows: (1) letters, reviews, case reports, conference abstracts, editorials, expert opinion and non-English language papers were excluded; (2) articles that had no information of OS or that could not calculated the HR about OS from the given information were excluded.

### Data extraction and critical appraisal

Data extraction was performed independently by two authors (CL and ZL) from eligible studies. Controversial problems were resolved by discussion and consensus. Two investigators reviewed all of researches that met inclusion and exclusion criteria. The first author name and publication year of article, cancer TNM (Tumor Nodal-involtment Metastasis), stage, clinicopathological parameters, immunohistochemical technique, survivin positive expression, and patient survival results from each study were documented.

### Assessment of study quality

Study quality was assessed independently by two investigators (CL and ZL), by means of reading and evaluating according to Newcastle–Ottawa quality assessment scale [11]. Briefly, the overall star assessed three main categories on the following: (1) selection of cohort; (2) comparability of cohort; and (3) ascertainment of outcome. A study can be awarded a maximum of one star for each numbered item within the Selection and outcome categories. A maximum of two stars can be given for Comparability. The total number of star was accumulated at last, with more stars reflecting a better methodological quality.

### Statistical analysis

We extracted and combined the data of survivin expression and clinicopathological parameters associated with ESCC from studies, and made a meta-analysis. Data combination included T1 and T2, T3 and T4, stage I and stage II, stage III and stage IV, well differentiation and moderate differentiation.

For quantitative evaluation of OS results, HR was used to estimate the impact of survivin expression on OS. HR and its variance for each individual study were extracted or calculated based on the published researches according to the methods described by Parmar [12]. Kaplan-Meier curves were read by Engauge Digitizer version 4.1 (http://digitizer.sourceforge.net/). Odds ratio (OR) was used to measure the relationship of survivin expression and clinicopathological features of ESCC. Heterogeneity was estimated by Cochran's test. A fixed-effect model was used when heterogeneity was not detected (P>0.10); otherwise, a random-effect model was used. All statistical analyses were performed by Review manager 5.0 (http://www.cochrane.org). A significant two-way *P* value for comparison was defined as *P*<0.05.

## Results

### Study characteristics

We found 85 studies with title which indicated they were potentially eligible for inclusion (Figure 1). After scrutinizing the abstracts and full-text articles of these studies, seven studies were deemed completely eligible for meta-analysis and their characteristics of seven eligible studies were summarized in Table 1. These studies that were published from 2003 to 2011 met the inclusion criteria for our meta-analysis [13]–[18]. Total 204 ESCC patients were employed to research the relationship between survivin expression and clinicopathological features or OS, but we could not obtain the information about survivin expression in nuclei or cytoplasm. Another 113 patients were employed to investigate the relationship between positive expression of survivin in cytoplasm and ESCC clinicopathological features or OS, and 277 patients were employed to investigate the association between positive expression of survivin in nuclei and ESCC clinicopathological features or OS.

Survivin expression was detected by immunohistochemistry in all publications and the immunohistochemical technique was summarized in Table 2. From Table 2, we knew that the immunohistochemical technique was varied widely among studies, with a wide range of dilution (1∶10–1∶100) and sources of primary antibodies coming from different companies.

### Methodological quality of the studies

For included studies, two authors independently extracted data and assessed methodological quality using the Newcastle–Ottawa quality assessment scale. Seven studies, with high levels of methodological quality (≥6 stars on the Newcastle–Ottawa scale) [19] were included in our meta-analysis.

### Impact of survivin expression on OS of ESCC patients

In this meta-analysis, we conducted 3 studies dealing with survivin expression and OS, including a total of 204 ESCC patients, yet survivin subcellular location was unclear. There was a significant heterogeneity among three studies (*P*<0.01), and thus a random effect model was used in meta-analysis. The pooled HR was 2.30 (95% CI: 1.51–3.51; Z = 3.86; *P* = 0.0001), illustrating that survivin expression was significantly with the worse OS of ESCC patients (Figure 2).

To further investigate the relationship between survivin subcelluar location and OS, two studies, which reported survivin expression was located in cytoplasm in 113 patients, were enrolled in this meta-analysis. Because of heterogeneity, a random effect model was adopted. The combined HR was 0.96 (95% CI: 0.96–5.69; Z = 0.04; p = 0.97), which illustrated that survivin expression in cytoplasm was not significantly associated with OS of ESCC patients (Figure 3).

We also enrolled 3 studies, which reported survivin was located in nuclei in 277 patients, to research the correlation between survivin expression and OS. Due to heterogeneity, a random effect model was accepted. The combined HR was 1.89 (95% CI: 1.45–2.96; Z = 4.69; *P*<0.0001), which demonstrated that positive expression of survivin in nuclei was significantly associated with poor prognosis of ESCC patients (Figure 4).

### Survivin expression and clinicopathological features of ESCC

Two studies evaluated the correlation of survivin expression in cytoplasm with stage of 113 ESCC patients. The combined OR was 0.36 (95% CI: 0.14–0.93; Z = 2.10; *P* = 0.04), without heterogeneity (*P* = 0.36), suggesting that survivin expression in cytoplasm was associated with advancement of ESCC (Figure 5). The combined OR for eligible studies that analyzed the relationship between survivin expression in cytoplasm and differentiation grade was 0.47 (95% CI: 0.05–4.55; Z = 0.65; *P* = 0.52), suggesting that positive expression of survivin had no significant effect on differentiation grade. We also founded that positive expression of survivin in cytoplasm had no correlation with lymph node status or metastasis. The pooled OR was 0.53 (95% CI: 0.21–1.32; Z = 1.36; *P* = 0.17), or 0.39 (95% CI: 0.39–3.06; Z = 0.17; *P* = 0.48), respectively (Table 3).

Also, there was no significant association between positive expression of survivin in nuclei and differentiation grade, lymph node status, depth of invasion, stage, or metastasis. The combined OR was 1.92 (95% CI: 0.73–5.05; Z = 1.31; *P* = 0.39), 0.51 (95% CI: 0.14–1.87; Z = 1.01; *P* = 0.31), 1.05 (95% CI: 0.24–4.65; Z = 0.06; *P* = 0.95), 0.58 (95% CI: 0.16–2.08; Z = 0.84; *P* = 0.40) or 0.93 (95% CI: 0.41–2.15; Z = 0.16; *P* = 0.87), respectively, indicating that survivin expression in nuclei had no significant impact on the clinicopathological features of ESCC patients (Table 4).

### Publication bias

Because the number of study included in our meta-analysis was comparatively few, we did not draw funnel plot to demonstrate publication bias.

## Discussion

Meta-analysis is a quantitative method to combine the results of randomized controlled trails. Recently, this approach has been used successfully for evaluation of prognostic indicators in patients with malignant diseases [20]–[23]. The study about relationship between survivin expression and clinicopathological features was comparatively few, and reports about prognostic significance of survivin in ESCC are controversial. Therefore, it is rather necessary to combine and analyze these data to reach a reasonable result. In the present study, we enrolled 7 studies concerning over-expression of survivin on ESCC clinicopathological features and patients' OS. In all studies, survivin expression was detected by immunohistochemistry with surgical specimens. By meta-analysis, survivin seemed to be a factor for poor prognosis in ESCC. Data from two studies, in which authors did not investigate the survivin subcellular location, were combined to show that survivin expression led to shorter OS in ESCC patients. We further analyzed another three studies, in which survivin expression was located in nuclei, the results showed that survivin expression in nuclei was closely associated with poor prognosis of ESCC patients. However, survivin expression in cytoplasm showed no significant impact on patients' OS. Besides, our results also showed that expression of survivin was associated with advanced stage of ESCC.

Survivin is the smallest member of the inhibitor of apoptosis protein (IAP) family, which contains a common hallmark that is a single baculovirus IAP repeat. Survivin possesses a lot of biological functions. Multiple studies have shown that survivin can inhibit apoptosis [24]. Also survivin plays an important role in promotion of mitosis in cancer cells. Even some researchers suggested that cell division control is a primary function of survivin [25]. In addition, some studies demonstrated that survivin has also been implicated in angiogenesis [26]–[28]. Survivin expresses highly in most human tumors and fetal tissues, but is undetected in most terminal differentiation cells [29]. Because of its selective expression, survivin is gradually regarded as a cancer prognostic hallmark and new target [30], [31]. Ikeguchi and Kaibara [32] observed that survivin expression positively correlates with the proliferative activity of ESCC cells and is an accurate prognostic marker for ESCC. Our results showed that nuclear expression of survivin had a significant impact on prognosis of ESCC patients, but cytoplasmic expression of survivin showed no prognostic relevance. Whereas, cytoplasmic expression of survivin was associated with advanced stage of ESCC, but nuclear expression of survivin was not. Thus, subcellular distribution of survivin was an important influence factor on clinicopathological features of ESCC.

In our meta-analysis, we had dealt with numerous heterogeneity problems. Heterogeneity is a potential problem to affect meta-analysis results. Although we chose these studies of only performing immunohistochemical staining to reduce heterogeneity as soon as possible, many reasons, such as primary antibodies from different companies, wide range of dilutions, different evaluation standards, length of follow-up, inconsistency of clinicopathological parameters, contributed to the heterogeneity. Accordingly, more objective methods are required to evaluate immunohistochemical results. Meanwhile, there are some limitations in this meta-analysis. First, we did not take into account unpublished articles and abstracts, because a lot of needed information can not be required. Second, we enrolled eligible English studies only so that there might be some biases because of excluding parts of qualified studies based on language criteria. Third, if we did not get HR from aricles directly, it was calculated from data or extrapolated from survival curves in the articles, the HR information obtained by statistical software unavoidably developed a decrease of reliability.

In conclusion, survivin over-expression in clinical tumor specimens was associated with a worse prognosis in patients with ESCC in our meta-analysis. Nuclear expression of survivin may be regarded as a prognostic factor for ESCC patients based on the currently obtained data. In contrast, survivin expression in cytoplasm was closely associated with advanced stage of ESCC patients. Larger clinical researches should be performed to investigate the precise prognostic significance of survivin, especially its subcellular location should be taken into account carefully.
